# Live imaging analysis of epithelial zippering during mouse neural tube closure

**DOI:** 10.1007/978-1-0716-2887-4_10

**Published:** 2023-01-01

**Authors:** Matteo A. Molè, Gabriel L. Galea, Andrew J. Copp

**Affiliations:** 1 Newlife Birth Defects Research Centre, Great Ormond Street Institute of Child Health, University College London, 30 Guilford Street, London, WC1N 1EH, UK; 2 Present address: Babraham Institute, Babraham Research Campus, Cambridge, CB22 3AT, UK; 3 Comparative Bioveterinary Sciences, Royal Veterinary College, London, NW1 0TU, UK

**Keywords:** Epithelial closure, neural tube, live imaging, cell shape, neural tube defects, tissue fusion

## Abstract

Zippering is a phenomenon of tissue morphogenesis whereby fusion between opposing epithelia progresses unidirectionally over significant distances, similar to the travel of a zip fastener, to ultimately ensure closure of an opening. A comparable process can be observed during *Drosophila* dorsal closure and mammalian wound healing, while zippering is employed by numerous organs such as the optic fissure, palatal shelves, tracheoesophageal foregut and presumptive genitalia to mediate tissue sealing during normal embryonic development. Particularly striking is zippering propagation during neural tube morphogenesis, where the fusion point travels extensively along the embryonic axis to ensure closure of the neural tube. Advances in time-lapse microscopy and culture conditions have opened the opportunity for successful imaging of whole-mouse embryo development over time, providing insights into the precise cellular behaviour underlying zippering propagation. Studies in mouse and the ascidian *Ciona* have revealed the fine-tuned cell shape changes and junction remodelling which occur at the site of zippering during neural tube morphogenesis. Here, we describe a step-by-step method for imaging at single-cell resolution the process of zippering and tissue remodelling which occurs during closure of the spinal neural tube in mouse. We also provide instructions and suggestions for quantitative morphometric analysis of cell behaviour during zippering progression. This procedure can be further combined with genetic mutant models (e.g. knockouts), offering the possibility of studying the dynamics of tissue fusion and zippering propagation, which underlie a wide range of open neural tube defects.

## 1 Introduction

Zippering is a key process of morphogenesis whereby two opposing epithelial sheets become progressively united over time, establishing new tissue connectivity. Similar to the travel of a zip fastener, the point of zippering is able to propagate over significant distances along an open organ or tissue, progressively bringing together the edges of two opposing epithelia to mediate fusion. The forward progression of zippering ensures the establishment of *de novo* epithelial continuity during embryonic development, or after an injury, ultimately leading to closure of an unsealed gap [1–3].

Epithelial zippering underlies closure of numerous organs during embryonic development, such as the neural tube [4], the optic fissure [5, 6], the palatal shelves [7], the tracheoesophageal foregut [8] and the presumptive genitalia [9]. Failure of zippering progression leaves the resulting organ unsealed, leading to severe open defects and congenital malformations such as neural tube defects, coloboma, cleft palate, tracheoesophageal fistula and hypospadias.

**Fig. 1:**
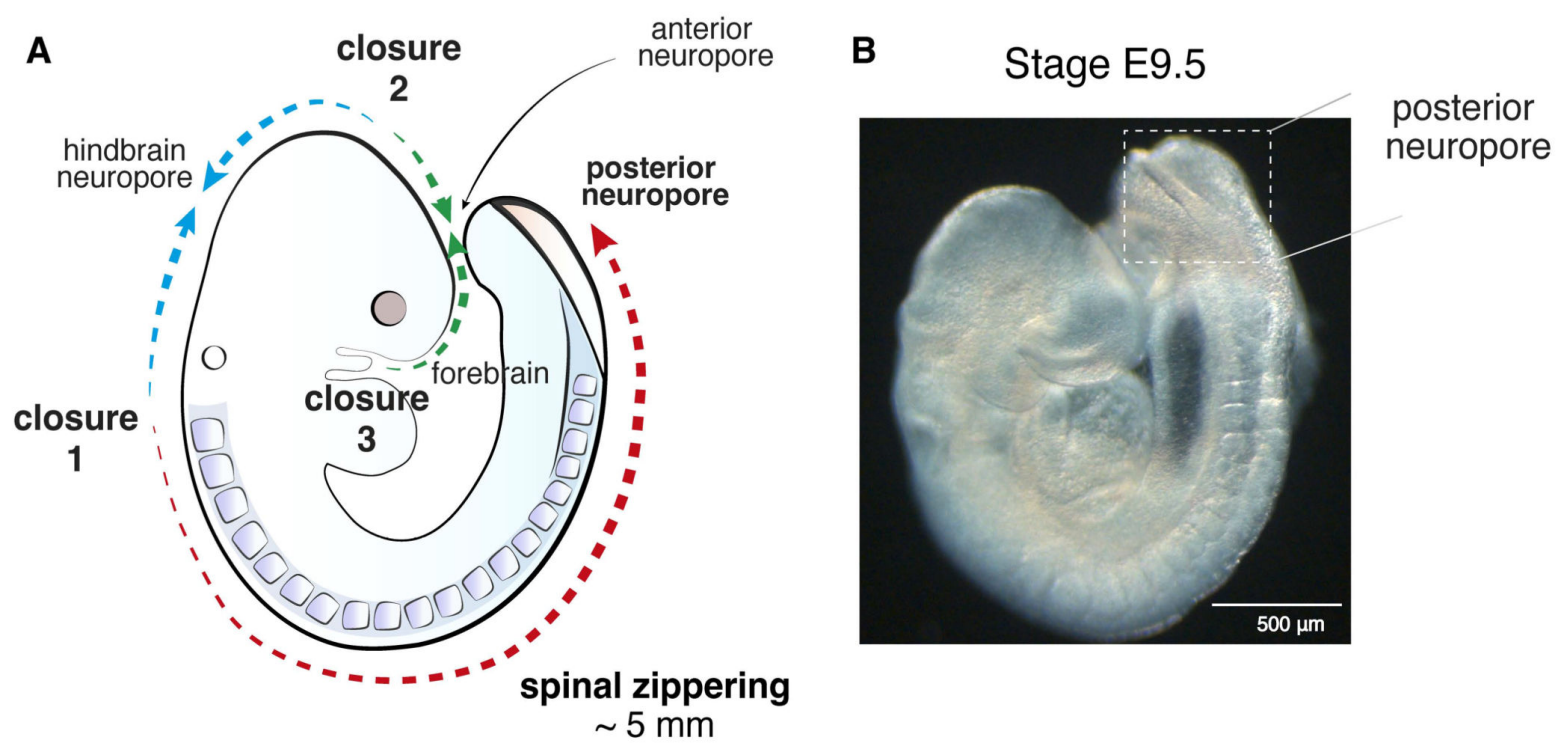
Zippering during mouse neural tube closure. (**A**) Schematic of zippering. Closure of the neural tube initiates at the boundary between the hindbrain and spinal cord (Closure 1). The wave of zippering then propagates bidirectionally. A second initiation site, Closure 2, occurs at the boundary between forebrain and hindbrain. This may be specific to mouse and probably does not occur in humans. Closure 3 forms at the very rostral end of the forebrain. Regions of open neural tube between sites of closure initiation are termed neuropores. In mouse, cranial closure completes by sealing of the anterior and, later, the hindbrain neuropore. Spinal closure progresses from Closure 1 along the caudal axis, until the entire open region of the posterior neuropore is eventually closed. Spinal zippering is estimated to cover a total length of approx. 5 mm to seal the caudal neural tube. (**B**) Representative brightfield image of mouse embryo at E9.5 with visible open posterior neuropore.

During closure of the vertebrate neural tube, zippering first originates in a region located at the boundary between the hindbrain and spinal cord, named Closure 1 (Fig. 1A), where the neural folds come into contact and fuse dorsally for the first time. From this site, zippering proceeds bidirectionally as rostrally and caudally-directed waves of fusion. In mouse and human embryos, Closure 1 initiates at embryonic day 8.5 (6 somite stage) and 18 days post-fertilisation, respectively [10]. Studies in chick [11], mouse [12, 13] and human embryos [14] have shown multiple *de novo* sites of closure initiation during neurulation, while zippering occurs between these sites to ensure closure along the entire body axis. While an additional closure emerges at the very rostral end in both mice and humans, named Closure 3, mouse embryos initiate fusion also at the midbrain-forebrain boundary, named Closure 2. However, this closure event is unlikely to occur in human embryos [14].

Zippering distances during closure of the cranial neural tube are relatively short, whereas caudally directed zippering, which originates from Closure 1, travels over a considerable distance (Fig. 1A, B), taking around 36 h to accomplish fusion of the entire posterior axis. This caudally directed progression of zippering eventually seals the last open region of neural tube, termed the posterior neuropore (PNP), thus completing the process of primary neurulation in mouse at the stage of 30 somites (E10) and in human embryos at 26-28 days post-fertilisation [15].

The primary cells involved in the process of epithelial zippering are the surface ectoderm cells, which are the first to mediate contact between opposing neural folds [16, 17]. At the site of fusion, these cells display intense protrusive activity, with filopodia at high spinal levels and ruffles (sheet-like lamellipodia) at low spinal regions [16]. Another major feature is the short proximal junctions, which cause these cells to adopt a wedge-shaped morphology and form an well-organised semi-rosette configuration around the site of zippering [18].

Live imaging of mouse neural tube closure is technically challenging, as the embryo needs to be maintained in an optimal culture system that allows continued normal development while being imaged under static conditions. Previously, *in toto* mouse embryo live imaging protocols have been developed [19] and more recently long-term culture optimisations using light-sheet microscopy were achieved [20, 21]. Here, we present a procedure developed to visualise and analyse the behaviour of the surface ectoderm cells at the site of zippering during closure of the mouse caudal neural tube. Using this method, we found that cells lining the open region of the posterior neuropore exhibit a broadly ‘rectangular’ morphology, characterised by long proximal junctions [18]. These become progressively shortened as the cells approach the site of zippering, acquiring a characteristic wedge-shape morphology around this site. Further proximal junctional narrowing causes cells to arrange into a semi-rosette configuration around the common vertex of the zippering point. This process brings cells from the two opposing sides of the neural folds into proximity, enabling cross-midline junction formation and forward propagation of zippering, as new cells bordering the open region undergo the same cycle of sequential constriction and semi-rosette formation. As the wave of zippering passes, surface ectoderm cells exit from the semi-rosette to overlie the most recently closed neural tube, now exhibiting a rostro-caudally elongated morphology [18].

In the present chapter, we provide details on how to explant and recover mouse embryos from the uterus, and how to mount the posterior region for visualization of zippering. Our method employs the fluorescent reporter line *Rosa26*^mTmG^ [22] for optimal cell visualisation, which can be used either unrecombined, thus imaging tdTomato fluorescence ubiquitously, or recombined by a suitable surface ectoderm-expressed Cre. Here we describe *Grhl3*-Cre-mediated recombination of the surface ectoderm cells at the site of zippering to express EGFP, enabling tissue specificity. Then we provide a step-by-step protocol for quantifying cell shape changes by manual segmentation. Our procedure can be adapted for both inverted and upright confocal microscopy, and used to image zippering progression at any axial level of the developing embryo. It can also be combined with genetic models of faulty neural tube closure, to investigate the effect of gene alteration on zippering progression.

## 2 Materials

### 2.1 Mouse lines

1. Fluorescent reporter line mTmG: *Rosa26*^mTmG^ [22] (gene symbol: Gt(ROSA)26Sor^tm4(ACTB-tdTomato,-EGFP)Luo^, Mouse Genomics Informatics (MGI): 3716464).
2. *Grhl3*-Cre line [23] (gene symbol: Grhl3^tm1(Cre)Cgh^, MGI: 4430902).

### 2.2 Embryo dissection

1. Dulbecco’s Modified Eagle’s Medium (DMEM).
2. Dissection medium: DMEM containing 25 mM HEPES (DMEM, stored at 4 °C) and supplemented with foetal bovine serum (FBS, heat-inactivated at 56 °C for 30 min). Dissection medium should be made fresh and pre-warmed to 37 °C before embryo collection and dissection. HEPES ensures buffering of the solution (pH 7.0), slowing its progressive alkalinisation during dissection in an ambient air environment. Embryos rapidly deteriorate in serum-free medium, and should not be dissected in DMEM alone or PBS.
3. Petri dishes: 60 mm.
4. Dissecting scissors and watchmakers’ forceps (number 5, Dumont, stainless steel). Forceps should be sharpened by gently stroking the tips on a sheet of fine emery paper, wiping clean of iron filings, and observing on a stereomicroscope. Repeat multiple times if necessary. The aim is to ensure the forceps meet precisely at their tips, enabling most accurate dissection. Use older forceps (coarser tips) for early dissection steps (e.g. removal of decidua from the uterus), and the newest forceps (finest tips) for later stages of dissection (e.g. removal of Reichert’s membrane). Sterilise dissecting instruments in 70% ethanol before starting dissection.
5. Stereomicroscope for the dissection procedure. This should have capacity for both darkfield and brightfield transmitted (under-stage) illumination. Early stages of dissection are most readily achieved using darkfield illumination, whereas we switch to brightfield for later stages when embryonic structures can be visualised through the yolk sac. Optional: the use of a heated stage fitted onto the stereoscope base helps to maintain optimal temperature control during embryo dissection.
6. Plastic pasteur pipettes. Cut the tip to ensure the opening is 1.5 times larger than the diameter of the membrane-enclosed embryos. This avoids damage while pipetting the embryos.

### 2.3 Embryo pre-culture

1. Rat serum is prepared as described previously [24, 25] and should be stored in aliquots (1–5 ml) at −20 °C and thawed immediately before use. Filter the serum by gently pushing from a syringe through a 0.45 μm pore size filter (Millipore). Pool all aliquots of serum for a given experiment, and then transfer equal volumes into 30 ml plastic culture tubes (Nunc). We culture 1-2 embryos per ml serum (E8.5 to E9.5), with 2-3 ml serum per tube. Seal culture tubes with a smear of silicone grease (Borer Chemie) on the lid surface that contacts the tube. Culture tubes are gassed with 5% O_2_, 5% CO_2_, 90% N_2_ for 1 min (for stage E8.5) embryos, or 20% O_2_, 5% CO_2_, 75% N_2_ (for stage E9.5). Gas the rat serum and warm in the roller incubator for 15 min before adding the embryos, after which tubes should be gassed again. Replenish gas environment at least every 12 h.
2. Roller culture incubator with 37 °C setting and ~ 30 rpm rotations. An alternative is to use a continuously-gassed rotator system. Both are available from Cullum Starr Engineering (http://www.cullumstarr.com/btc-engineering/precision-incubator).

### 2.4 Embryo mounting

1. To prepare agarose plates for embryo positioning, use regular melting point agarose at molecular biology grade. Microwave (800 W) 4% agarose powder in PBS, swirling intermittently to prevent excessive bubbling. Continue until the agarose is fully dissolved and air bubbles have dissipated. Pour 3 ml of solution into each 35 mm dish and allow to solidify.
2. Corning tissue culture dishes, 35 mm, for the agarose layer and embryo immobilisation. If using an inverted microscope, use glass-bottomed dishes suitable for high-resolution imaging from inverted objectives (Ibidi, glass-bottom dish 35 mm, Cat.No 81218-200). See Note 1.
3. Micro-surgical needles, purchased as swaged needles on 11–0 Mersilene (TG140-6; Ethicon) and 10–0 Prolene (BV75-3; Ethicon). Cut away and discard the suture material.
4. Imaging medium: 50% DMEM (containing 25 mM HEPES) and 50% rat serum. This is used for static culture during the live imaging process. Equilibrate dishes with imaging medium in a tissue culture incubator at 37 °C, gassed with 20% O_2_, 5% CO_2_, 90% N_2_, before transferring in the embryos.

### 2.5 Live imaging and analysis

1. Confocal microscope with temperature-controlled chamber at 37 °C and with humidification. For this protocol we use a Zeiss Examiner LSM880 microscope with a 20x (numerical aperture NA1) W-Plan Apochromat water dipping objective (working distance 3.7 mm). The method described is optimised for an upright microscope but can be adapted for inverted microscopy (see Note 1).
2. Fiji and associated plugs-ins [26]. Open access software can be downloaded from https://imagej.net/software/fiji/.
3. StackReg plugin for Fiji allowing registration in 2D and 3D: available at http://bigwww.epfl.ch/thevenaz/stackreg/ (for 2D) and https://imagej.net/plugins/correct-3d-drift (for 3D).
4. Surface subtraction macro [27] courtesy of Dr Dale Moulding, GitHub (https://github.com/DaleMoulding/Fiji-Macros).
5. For 3D cell segmentation, we suggest using Imaris: https://imaris.oxinst.com.
6. For deconvolution we suggest using Huygens: https://svi.nl/Huygens-Confocal-Software.

### 2.6 Whole-mount immunofluorescence

1. PBS: at 1X working concentration, this contains 137 mM NaCl, 2.7 mM KCl, 8 mM Na_2_HPO_4_, and 2 mM KH_2_PO_4_.
2. 4% ice-cold paraformaldehyde (PFA) in PBS for embryo fixation
3. Antibodies. Anti-E-cadherin either: Invitrogen/Thermo Fisher 13-1900, at 1:200 dilution, or BD Biosciences BD610182, at 1:200. Anti-zonula occludens 1 (ZO-1): Thermo Scientific 33-9100, at 1:200.
4. Permeabilization solution: PBS containing 0.3% Triton X-100 and 0.1 M glycine. Solution stored at 4 °C.
5. Blocking and antibody solution: PBS containing 10% FBS (heat inactivated at 56 °C for 30 min), 1% bovine serum albumin (BSA; filtered), and 0.1% Tween-20. This solution can be pre-made and aliquots stored frozen.
6. Washing solution: PBS containing 0.1% Tween-20.

## 3 Methods

### 3.1 Mouse lines and timed matings

1. To visualise embryonic cell dynamics over time, use the floxed reporter mouse line *Rosa26*^mTmG^, which expresses membrane-targeted tdTomato in all cells of the embryo. Live imaging can be performed either using un-recombined embryos expressing tdTomato fluorescence only or by inducing conversion of tdTomato (mT) into membrane-targeted EGFP (mG) to allow visualisation of both non-recombined (tdTomato+ cells) and recombined tissues (EGFP+ cells), respectively (see Note 2 for further explanations and rationale). Below, we describe the procedure using only non-recombined embryos expressing tdTomato.
2. Cross homozygous *Rosa26*^mTmG/mTmG^ mice to generate homozygous embryos (*Rosa26*^mTmG/mTmG^) for stable expression of membrane tdTomato fluorescence in all cells of the embryo.
3. Use mice aged 6 to 20 weeks with an average weight of 30 g. Set mice up for timed mating in the late afternoon and check the following morning for the presence of a copulation plug, designated embryonic day (E) 0.5.

### 3.2 Embryo dissection

1. Sacrifice pregnant females by cervical dislocation at the following gestational stages:
  • At E8.5 - embryos that have initiated closure 1 and started zippering;
  • At E9.5 - embryos that are undergoing zippering of the posterior neuropore;
  • At E10.0 - embryos at the stage of closure of the posterior neuropore.
  The procedure below describes the process for embryos at E9.5.
2. Remove the uterine horns from the pregnant female and dissect them in a petri dish of pre-warmed ‘dissection medium’. For a detailed account of the dissection procedure, see [24]. In brief, on the stage of a stereoscope, use scissors and a pair of sharpened forceps to trim away the blood vessels and fat from the mesometrial surface of the uterus. Then, gradually dissect open the uterus from the mesometrial surface of each implantation site in turn, taking care to minimise mechanical stress on the decidual swelling as it emerges from the uterine wall (hint: keep the tips of your two pairs of forceps close together). From E9.5 onwards, any increased pressure inside the implantation site can lead to the embryo being ejected from the uterus, leading to damage and unsuitability for culture. In this way, remove each decidual swelling and transfer to a fresh dish of dissection medium.
3. Dissect open each decidua swelling, beginning at the former mesometrial end (the wide, ‘fluffy’ surface). The aim is to gently open the decidua and peel out the embryo with its covering membranes (i.e. the conceptus). At this point, the embryo is covered with trophoblast that is adherent internally to the thin, elastic Reichert’s membrane. Inside this is the yolk sac, which encloses the amnion that immediately surrounds the embryo. The trophoblast is thickened at one embryonic pole, forming the ectoplacental cone.
4. Transfer the dissected conceptuses to a fresh dish of pre-warmed dissection medium using a plastic pasteur pipette, cut to a suitable orifice size. Next, remove the trophoblast/Reichert’s membrane layer, a procedure that requires careful dissection with the finest forceps. Gently pinch the trophoblast and Reichert’s membrane using both pairs of forceps and tear apart carefully. If Reichert’s membrane ruptures, you will see the embryo and yolk sac bulging through the opening you have created. The trophoblast/Reichert’s membrane will remain as an intact sheet. Enlarge the opening so the embryo is fully exposed, taking care not to damage the yolk sac layer. Then trim off and discard the trophoblast/Reichert’s membrane layer from around the circumference of the ectoplacental cone. If Reichert’s membrane fails to rupture initially, you will find only small pieces of trophoblast become detached. Continue to gently pinch with both pairs of forceps until Reichert’s membrane ruptures. The conceptus is now ready for culture, consisting of the embryo enclosed in its amnion and surrounded by an intact external layer of yolk sac (rich in blood vessels), with the ectoplacental cone at the pole opposite to the embryo.

### 3.3 Pre-culture of embryos in rolling conditions

1. Transfer the dissected E9.5 embryos into 30 ml plastic culture tubes (Nunc), sealed with silicone grease, using 100% rat serum as culture medium, pre-gassed with 20% O_2_, 5% CO_2_, 90% N_2_ and pre-warmed to 37 °C. Try to carry over a minimum of dissection medium into the rat serum. See section 2.3.1 above for further details.
2. Incubate the embryos in rolling or rotating culture (see Section 2.3.2) for a minimum of 2 h. This ensures that embryos recover after the dissection procedure, before initiation of live imaging. Optimal viability of the embryos can be assessed by the presence of a continuous heartbeat and vigorous blood circulation through the yolk sac vessels.

### 3.4 Preparation for live imaging

1. Prepare an agarose plate (4% agarose in PBS) using a 35 mm dish. Once solidified, make some holes through the agarose using the blunt end of a p200 pipette tip. The size of the holes should be adjusted to accommodate the embryos that are to be imaged.
2. Rinse dishes with DMEM and add 2 ml of ‘imaging medium’ per dish. Equilibrate the dishes in an incubator at 37 °C, with a gas atmosphere of 20% O_2_, 5% CO_2_, 90% N_2_.
3. Prepare the confocal microscope (see section 2.5 above) and ensure that the environmental chamber is set to 37°C, gassed with 20% O_2_ (atmospheric), 5% CO_2_ and 90% N_2_ (atmospheric) and is constantly humidified with water to prevent medium evaporation. Allow at least 1 h for the chamber to equilibrate before use.

### 3.5 Embryo mounting for static culture

1. Retrieve one embryo from the rolling culture and transfer it into a dish containing pre-equilibrated ‘imaging medium’. All other embryos should be kept in the rolling culture, until needed for imaging.
2. Place the dish on the stage of a stereoscope and ensure optimal viability by confirming the presence of a continuous heartbeat and vigorous blood circulation through the yolk sac vessels.
3. Using the finest forceps, make a small ‘window’ through the yolk sac and underlying amnion to expose the dorsal surface of the posterior neuropore. At E9.5, the neuropore is situated close to the junction of yolk sac and ectoplacental cone. This allows the opening to be made near to this junction, where the yolk sac is least vascularised, hence avoiding damage to large vessels and excessive bleeding, which will otherwise adversely affect overall viability of the embryo in culture. For an alternative mounting procedure see Note 3.
4. Transfer the ‘windowed’ embryo into an agarose dish containing pre-equilibrated ‘imaging medium’. Move the embryo into one of the holes previously made in the agarose layer to partially immobilise it. Position the embryo so that the site of zippering (visible through the window) is oriented parallel to the surface of the medium, thus facing the observer (when using an upright microscope). For the procedure using an inverted microscope, see Note 1.
5. To ensure immobilisation of the embryo in the oriented position, insert microsurgical suture needles (see section 2.4.3 above) into the conceptus: one is placed through the allantois into the underlying agarose to immobile the caudal region. A second needle is placed around the embryonic body to prevent rotation and displacement during imaging. If the heart-beat interferes with imaging, additional needles should be added to support immobilisation of the region of interest. See Note 4 for further details on mounting.
6. Very carefully mount the dish containing the embryo in the microscope chamber, to start confocal imaging. Ensure the correct orientation of the zippering region has been maintained after transfer of the dish.

### 3.6 Live imaging of the zippering region

Here we describe the parameters for live imaging using an upright Zeiss Examiner LSM880 confocal microscope with a 20x (numerical aperture 1) and W-Plan Apochromat dipping objective (working distance 3.7 mm).

1. Manually re-adjust the field of view in x, y and z before acquisition of each z-stack by re-centering around the site of zippering. Displacement of the region of interest out of the field of view is very likely, despite the immobilisation procedures, due to embryo movements and heartbeat.
2. Parameters for imaging: keep laser power below 0.8% (488 nm wavelength to image membrane GFP or 561 nm for membrane tdTomato); gain can be modulated for optimal visualisation without over-exposure; open the pinhole to 1.86 AU (2.8 μm section); set z-size to 1.4 μm thickness (which takes approximately 50 slices for imaging the entire caudal region of the embryo), bidirectional imaging, resolution at 1024x1024 pixels, maximum speed, frame averaging: 2, format 8-bit.
3. Acquire each z-stack at an interval between 5 and 7 min. This procedure allows visualisation of zippering progression and cellular rearrangements as previously described, which occurs within 30 min [18]. A maximum period of 5 h continuous imaging can be achieved with the above procedure but embryo viability decreases significantly after a few hours due to continuous exposure to lasers (inducing phototoxicity), static culture conditions and exposure of the embryo directly to the culture medium due to opening of the yolk sac/amnion membranes. Another limiting factor is the photobleaching of tdTomato fluorescence which occurs over time.
4. Remove the dish from the incubation chamber after imaging and assess the viability of the embryo, which should exhibit continued heartbeat and yolk sac blood circulation. We discard imaging of any embryo that shows lack of heartbeat and/or poor circulation, as this likely indicates sub-optimal embryo development during the imaging period.
5. If desired, post-imaging embryos can be rinsed in ice-cold PBS and then immersed in fixative. Use ice-cold 4% PFA (in PBS) and incubate the samples overnight at 4 °C for optimal fixation. Samples should be then washed in PBS and stored at 4 °C: e.g. for immunofluorescence (see section 2.6)

### 3.7 Post-acquisition processing

**Fig. 2:**
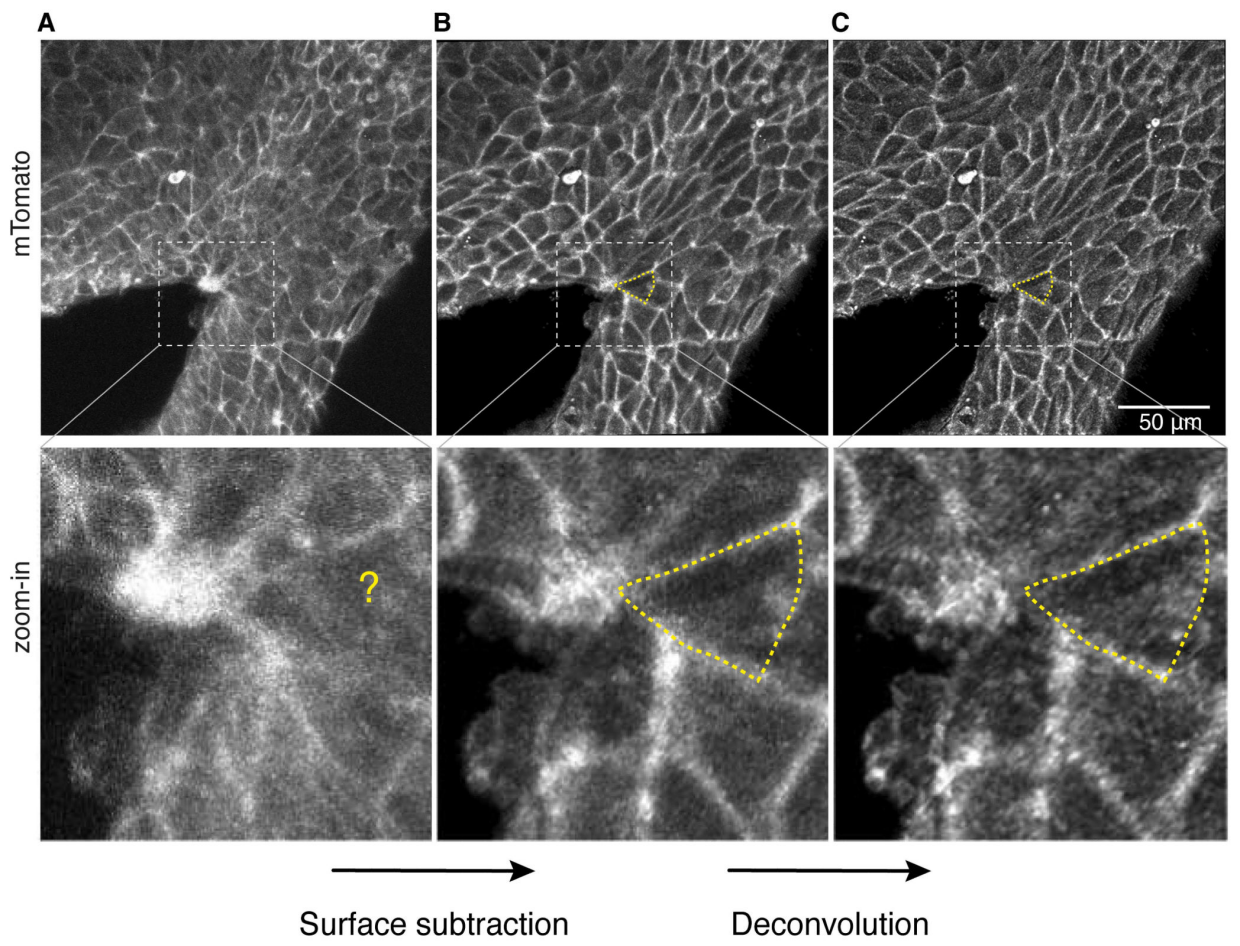
Post-acquisition processing steps. (**A**) Images, after initial acquisition, tend to be noisy, limiting the ability to clearly identify the precise boundary of each cell. (**B**) Digital surface subtraction of the surface ectoderm from the underlying neural folds further improves signal and contrast. (**C**) Deconvolution processing significantly improves image resolution by increasing signal and correcting noise, allowing easier segmentation for subsequent analysis.

Post-imaging processing is critical to extract quantifiable data and improve image resolution from the initial raw data acquired, which have low-intensity and high noise-to-signal ratio (Fig. 2A).

1. Correct 3D drift between time points using Fiji [26] and the associated plug-in https://imagej.net/plugins/correct-3d-drift.
2. Digitally dissect the signal of the surface ectoderm from the underlying neural folds (Fig. 2B) by using our previously reported surface subtraction macro [27] available at https://github.com/DaleMoulding/Fiji-Macros. This surface subtraction step allows selective visualisation of the most superficial cell layer corresponding to the surface ectoderm, digitally removing the signal from the neuroepithelium beneath.
3. Label each z-stack file and order by the time point of acquisition (e.g. t-1). Process the stack for each time point by z-projection using the max intensity projection option. Adjust brightness and use outlier removal option. To optimise the signal-to-noise ratio, images are processed by deconvolution using Huygens (Fig. 2C).
4. Combine the individual time point projections into a single stack (“*Image*” > “*Stack*” > “*Images to Stack*”).
5. To further correct for drift between the maximum projections, use the plug-in StackReg available at http://bigwww.epfl.ch/thevenaz/stackreg/ which allows registration of time points in 2D.

### 3.8 Segmentation and morphometric analysis of zippering

**Fig. 3:**
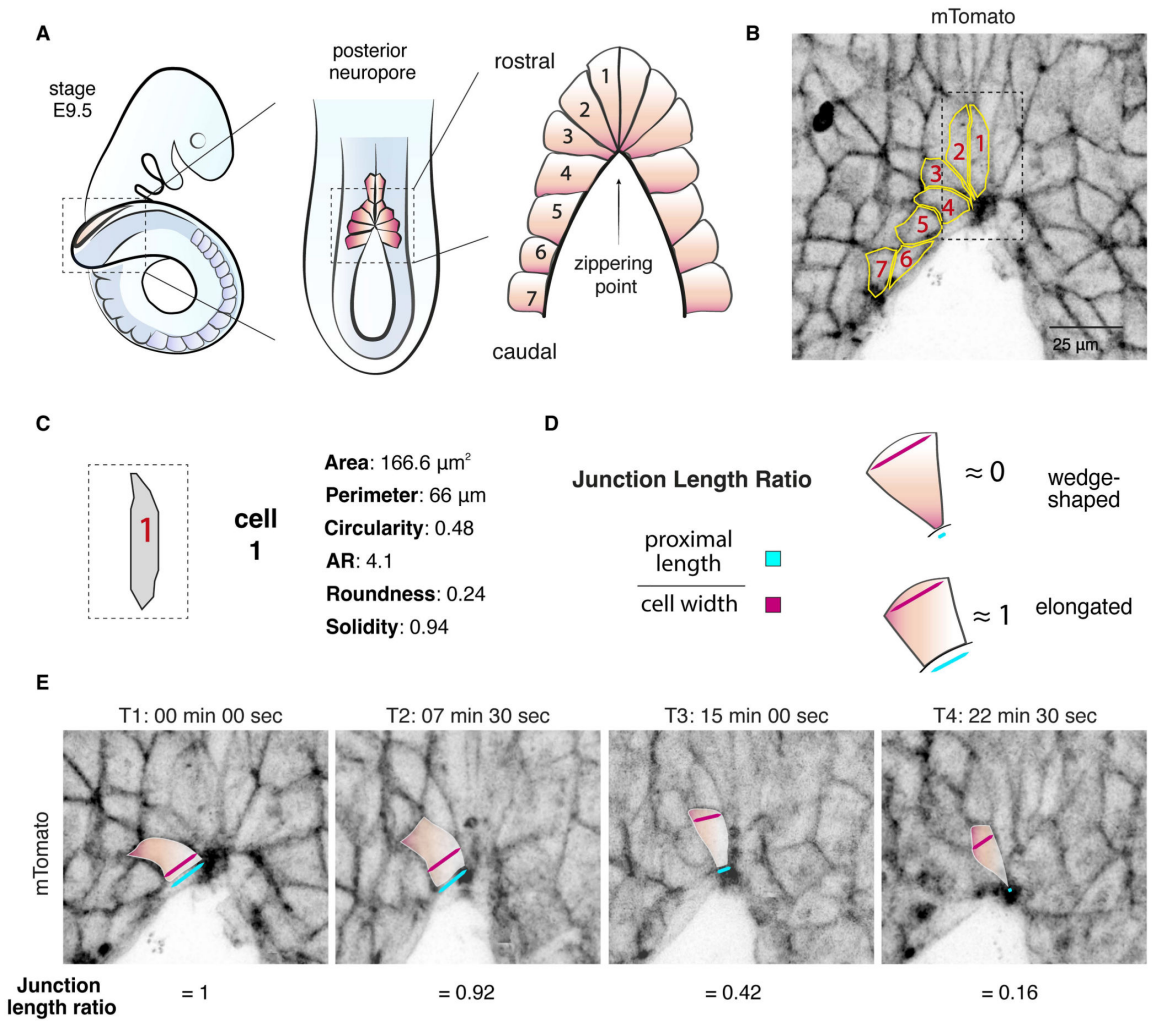
Segmentation and morphometric analysis of zippering. (**A**) Schematics of cell arrangements at the site of zippering (dorsal view). Surface ectoderm cells 1-3 display a wedge-shaped morphology. Cells bordering the open PNP (cells 4–7) exhibit a more rectangular morphology. (**B**) Representative image of an un-recombined embryo expressing tdTomato fluorescence (*Rosa26*^mTmG/mTmG^) and manual segmentation of the cells at the site of zippering. (**C**) Morphometric analysis of cell 1. (**D**) Junction measurements and cell shape classification. Junction length ratio is calculated by dividing the length of the proximal junction by the cell maximum width. A junction length ratio ≈0 indicates a wedge-shaped morphology; a ratio ≈1 indicates an elongated morphology. (**E**) Stills from a live imaging movie showing cell shape changes during zippering. Cells shorten their proximal junctions over time to form a semi-rosette configuration at the zippering point.

1. To analyse the morphology of the surface ectoderm cells around the zippering point, use the polygon selection tool of Fiji for manual segmentation. Alternative methods for 3D segmentation are discussed in Note 5.
2. Number cells, from the closest to site of fusion (cell 1) to the one farthest caudally along the open fold (cell 7) (Fig. 3A) [18]. Repeat the process for each side of the open posterior neuropore.
3. Using the polygon selection tool, draw precisely around the perimeter of each cell with multiple points to achieve maximum possible accuracy (Fig. 3B). Optional: use the fit spline option (”*Edit*” > ”*Selection*” > “*Fit spline*”) to smooth the cell borders and ensure that the contour is a close match to the border of the cell. Add each cell selection to Region Of Interest (ROI) manager (“*Analyse*” > ”*Tools*” > “*ROI manager*”).
4. Before proceeding, make sure that the scale of the image is set up correctly. Usually, the scale is automatically recorded in the image's metadata when acquired by confocal microscopy. Therefore, you can check by pressing “*Analyse*” > “*Set Scale*” to retrieve this information. It will report the pixel/μ conversion, if correctly calibrated. Alternatively, the scale will need to be set up manually.
5. Use the set measurements (analyse, set measurement) to include the following parameters for the morphometric analysis of each selection (Fig. 3C): area (μm^2^), perimeter (length of the selection in μm) and shape descriptors. The latter calculates the following shape parameters: Circ. (circularity): 4π*area/perimeter^2 with a value of 1.0 indicating a perfect circle and a value approaching 0.0 indicating a highly non-circular elongated shape; AR (aspect ratio): major axis/minor axis of the best fit ellipse; Round (roundness): 4*area/(π*major_axis^2); Solidity: area/convex area. Press measurement to retrieve all the morphometric parameters for each segmented cell.
6. To determine whether the cell adopts a wedge-shaped morphology, calculate the junction length ratio by dividing the length of the proximal junction by the cell width (maximum width) (Fig. 3D). A junction length ratio ≈1 indicates an elongated morphology whereas a junction length ratio ≈0 indicates a wedge-shaped morphology, due to extreme shortening of the proximal junction compared with the width.
7. Repeat this quantification from cell 1 (close to zippering) to cell 7 (furthest from zippering into the open region) and for left and right sides of the open posterior neuropore. Cells shorten their proximal junctions and adopt a characteristic wedge-shaped morphology in dorsal view as they approach the site of zippering (cells 1-3). In contrast, cells that have not yet entered the zippering, bordering the open PNP, display an elongated morphology (cells 4-7).
8. Repeat the quantification described above for each cell at each time point (Fig. 3E). Shorter image intervals during acquisition (< 5 min) allow better identification and easier tracking of individual cells over time. However, this is likely to result in increased phototoxicity, affecting embryo viability and capability to image for a longer period. Over time, cells narrow their proximal junctions and become arranged into a semi-rosette configuration around the zippering point. This brings opposing junctions in proximity, enabling zippering propagation. This normal cell behaviour can be contrasted with mutant or drug-treated embryos that may exhibit abnormal cell behaviour during faulty zippering [18].

## 4 Notes

1. If using an inverted microscope, glass-bottomed plates (described in section 3.4) containing agarose can be used to ensure correct embryo orientation. After dissection and opening of the yolk sac and amnion membranes, the embryo should be gently inserted into the agarose hole, orienting the caudal region towards the bottom of the dish. It is essential to: (i) achieve good immobilisation of the embryo by placing the embryos in custom-made holes of suitable size in the agarose plate, and with additional stabilisation using microsurgical suture needles; (ii) ensure that the caudal region and the site of zippering is oriented parallel and close to the bottom surface of the plate.
2. To induce efficient recombination of surface ectoderm cells at the site of zippering, cross the *Rosa26*^mTmG/mTmG^ reporter line with the *Grhl3*-Cre driver [23]. Homozygous *Rosa26*^mTmG/mTmG^ mice, usually females, are mated with heterozygous *Grhl3*^Cre/+^ mice, usually males. Half the embryos will be doubly heterozygous (*Rosa26*^mTmG/+^; *Grhl3*^Cre/+^) and will express membrane EGFP at the site of zippering. The remaining embryos will express membrane tdTomato only (*Rosa26*^mTmG/+^ and *Grhl3*^+/+^). The success of Cre-mediated recombination and conversion from tdTomato into EGFP can be easily assessed in the freshly dissected embryos using a fluorescence stereomicroscope. The tdTomato fluorescence signal is of lower intensity than EGFP after Cre-mediated recombination at the *Rosa26*^mTmG^ locus. This approach offers the following advantages: (i) laser power can be reduced during live imaging; (ii) precise targeting of surface ectoderm cells is enhanced, by virtue of their EGFP expression when using Grhl3-Cre, both at the zippering point and bordering the open posterior neuropore; (iii) post-acquisition processing, such as surface ectoderm subtraction, is enhanced due to the stronger signal. It is important to note that in addition to the surface ectoderm cells, *Grhl3*^Cre/+^ induces recombination of some cells of the dorsal neuroepithelium, as we previously reported [16, 27].
3. An alternative mounting protocol to the one described here has been described by the Niswander group [19]. This is based on immobilisation of the embryo on a filter paper during static culture and live imaging.
4. Orientation and immobilisation of the embryo in the agarose well is essential for successful imaging of the zippering point, as achieved by the use of agarose wells and further stabilisation using inserted needles. The PNP and in particular the site of zippering need to be oriented perpendicular to the field of view of the objective to minimise curvatures, so as to ensure capture of optimal dorsal view images of the ROI, which is essential for the later segmentation step.
5. 3D segmentation is preferable but also more challenging. Here, we describe a 2D method due to the relatively flat squamous morphology of the surface ectoderm cells around the site of zippering. Imaris software offers a friendly pipeline for 3D cell segmentation, and an option also for tracking in 3D, but the costs of accessing this software are significant. Alternative options are also offered by Fiji or Icy. We suggest processing files for 3D drift correction (to account for any displacement over time) before starting segmentation.

## Supplementary Material

Movie 1 **Movie 1**: Live imaging sequence (37.5 mins) of cellular semi-rosette formation in the surface ectoderm during zippering at the mouse posterior neuropore (E9.5).
